# Cross-modal congruency modulates evidence accumulation, not decision thresholds

**DOI:** 10.3389/fnins.2025.1513083

**Published:** 2025-02-20

**Authors:** Natálie Brožová, Lukas Vollmer, Björn Kampa, Christoph Kayser, Janina Fels

**Affiliations:** ^1^Institute for Hearing Technology and Acoustics, RWTH Aachen University, Aachen, Germany; ^2^Systems Neurophysiology Department, Institute of Zoology, RWTH Aachen University, Aachen, Germany; ^3^Department of Cognitive Neuroscience, Universität Bielefeld, Bielefeld, Germany

**Keywords:** multisensory perception, audio-visual integration, perceptual decision-making, cross-modal correspondences, EEG, cognitive modeling

## Abstract

Audiovisual cross-modal correspondences (CMCs) refer to the brain's inherent ability to subconsciously connect auditory and visual information. These correspondences reveal essential aspects of multisensory perception and influence behavioral performance, enhancing reaction times and accuracy. However, the impact of different types of CMCs–arising from statistical co-occurrences or shaped by semantic associations–on information processing and decision-making remains underexplored. This study utilizes the Implicit Association Test, where unisensory stimuli are sequentially presented and linked via CMCs within an experimental block by the specific response instructions (either congruent or incongruent). Behavioral data are integrated with EEG measurements through neurally informed drift-diffusion modeling to examine how neural activity across both auditory and visual trials is modulated by CMCs. Our findings reveal distinct neural components that differentiate between congruent and incongruent stimuli regardless of modality, offering new insights into the role of congruency in shaping multisensory perceptual decision-making. Two key neural stages were identified: an Early component enhancing sensory encoding in congruent trials and a Late component affecting evidence accumulation, particularly in incongruent trials. These results suggest that cross-modal congruency primarily influences the processing and accumulation of sensory information rather than altering decision thresholds.

## 1 Introduction

Perception is not limited to information derived from a single sensory modality; rather, it involves the integration of multisensory inputs, which collectively inform and refine our perceptions (Franzen et al., 2020; Mercier and Cappe, 2020; Romo and de Lafuente, 2013). A key aspect of multisensory integration is the presence of cross-modal correspondences (CMCs)—associations between stimuli across different sensory modalities—that help guide accurate and efficient decision-making (Bizley et al., 2016; Tuip et al., 2022).

Audiovisual CMCs refer to consistent associations that our brain establishes between auditory and visual modalities. For example, a high-pitched sound is naturally linked to a small visual object, while a low-pitched sound is linked to a larger visual object (Bien et al., 2012; Spence, 2011; Gallace and Spence, 2006; Sciortino and Kayser, 2023). These cross-modal correspondences reveal the underlying ways in which our brain interprets sensory information. However, different CMC types—statistical, structural, or semantically mediated—may have varying consequences for human decision-making and information processing and might originate from different neural mechanisms (Spence, 2011; Spence and Parise, 2012). Statistical correspondences arise from the frequent co-occurrence of sensory features in the environment, such as the pairing of large objects with low-pitched sounds (Deroy et al., 2013; Spence and Parise, 2012; Parise and Spence, 2012). Structural correspondences are based on inherent similarities between the sensory properties of different modalities (Spence, 2020), such as the association between sharp sounds and angular shapes. Semantically mediated correspondences, in contrast, involve learned associations influenced by language (McCormick et al., 2018; Spence, 2011), such as the association between high-pitched sounds and higher spatial positions or low-pitched sounds and lower spatial positions. These metaphorical mappings are often encoded in language, like describing a “high note” or a “low tone,” but they also rely on universal perceptual experiences (Eitan and Timmers, 2010). It is important to note that these types are not entirely distinct. For instance, statistical components may contribute to semantic correspondences (e.g., size associations in language), and semantic interpretations may reinforce statistical pairings through frequent co-occurrence (Spence, 2011). These cross-modal associations significantly influence perceptual decision-making by altering how sensory information is processed and interpreted. Audiovisual CMCs often rely on relative, rather than absolute, sensory attributes. For example, congruency effects are shaped by the relative pitch of auditory stimuli (a perceptual attribute derived from the physical property of frequency) and the relative size or elevation of visual stimuli (perceptual qualities linked to physical dimensions such as diameter), rather than their absolute values (Spence, 2019). This relative nature of congruency highlights the brain's ability to interpret sensory input within context-dependent frameworks, which is essential for guiding perceptual decision-making in varying environments. Behavioral performance improvements, such as faster response times (RTs) and higher accuracy, have been demonstrated for congruent cross-modally associated stimuli (Parise and Spence, 2012; Kayser and Kayser, 2018; Franzen et al., 2020; Kayser et al., 2017; Tuip et al., 2022). Understanding these effects is essential for uncovering the mechanisms that underlie efficient sensory processing and perceptual judgment.

In particular the origin and neuro-functional correlates of CMCs remain debated and previous work disagrees on whether they result in low-level sensory cortices or whether they are the result of high-level integration processes in semantic or object identification networks in associative cortices (McCormick et al., 2018; Sciortino and Kayser, 2022). Some studies support a low-level origin: for example an EEG study on sound-symbolic associations found association-specific activations around 140 ms (Kovic et al., 2010) and primary auditory and visual cortices are activated in the Bouba-Kiki effect (Peiffer-Smadja and Cohen, 2019) or other multisensory paradigms (Brang et al., 2022; Kayser et al., 2010; Lakatos et al., 2009; Schroeder and Foxe, 2005). Other studies, however, support a high-level origin in the parietal and frontal regions. These include Some EEG studies on the pitch-size association (Bien et al., 2012; Stekelenburg and Keetels, 2016), and fMRI studies on cross-modal associations (McCormick et al., 2018, 2021). Finally, one EEG study that is methodologically related to the present work (Bolam et al., 2022) examined EEG responses to auditory trials for CMCs between auditory pitch and size found early sensory (around 100 ms post-stimulus onset) and late decisional (300–400 ms post-stimulus onset) components that distinguish between congruent and incongruent audiovisual pairings. Hence, across the literature, there does not seem to be a consistent picture of whether cross-modal associations reflect more late and decision-related processes or indeed engage early and low-level sensory processes.

Our study aims to deepen the understanding of the neural origins of audiovisual cross-modal associations. We focus on two distinct types of CMCs statistical and semantic examining whether the underlying processes observed in previous research are confined to specific modalities and CMC types, or whether they generalize across sensory systems. By investigating these different types of congruency, we aim to uncover modality-independent mechanisms that govern how congruency influences perceptual or decision processes across various sensory contexts.

For our study, we combined the implicit association test (IAT) (Parise and Spence, 2012) with concurrent EEG recordings. In this IAT, cross-modal correspondences are probed in unisensory trials via different stimulus-response assignments presented in different blocks. Hence, the cross-modal congruency is manipulated by altering stimulus-response mappings between blocks. Importantly, the design avoids confounding influences of selective attention arising in experiments where multiple stimuli are presented in each trial and avoids tapping into processes that judge the spatio-temporal congruency of simultaneously presented stimuli, which are other factors relevant to multisensory integration that do not directly pertain to CMCs. This contrasts the present study from previous work, which often relied on speeded classification tasks (Marks, 2004; Bien et al., 2012).

By using the IAT, we were able to isolate the specific effects of audiovisual congruency on perceptual decision-making. Combining the IAT with EEG, multivariate analysis, and neurally informed cognitive modeling (Bolam et al., 2022; Franzen et al., 2020; O'Connell et al., 2018), our approach provides insights into the neural stages of processing where congruency effects between acoustic and visual stimuli occur, and how these effects shape perceptual decision-making. This methodology enables us to examine the neural dynamics underlying cross-modal associations and their role in decision formation across different types of CMCs.

## 2 Materials and methods

### 2.1 Participants

Thirty participants (14M, 16F, aged 23–32, mean 27,3 years old) joined this study, all with normal/corrected vision, and hearing and no self-reported history of neurological disorders. Participants were multilingual, with diverse linguistic backgrounds. Experiment instructions were in English. Given the abstract nature of the audiovisual stimuli used in this study, linguistic influences on semantic processing were considered minimal. They received €15/h for their participation. The experiment adhered to the ethical guidelines outlined in the Declaration of Helsinki. Prior to the experiment, all participants gave written informed consent to participate and agreed to the processing of their data in accordance with EU data protection regulations.

### 2.2 Stimuli and apparatus

Two auditory and two visual stimuli were used for each experiment. Auditory stimuli consisted of two 300 ms tones (“high” and “low” pitch, 2,000 Hz and 100 Hz respectively). Visual stimuli consisted of two dark gray circles [“small” and “large”, 1 and 5 height units respectively, as defined in PsychoPy (v. 2023.1.3) (Peirce et al., 2019)] for size CMC and of two dark gray circles (“high” and “low”, 6 and –6 height units relative to monitor center respectively) for elevation CMC (see task description). Visual stimuli were also presented for 300 ms. All stimuli were created and presented using Python (v. 3.11) and PsychoPy (v. 2023.1.3) (Peirce et al., 2019). The experiment was conducted in an acoustically isolated hearing booth. Both tones were calibrated to a loudness of 4 sone, which approximately corresponds to 60 dB SPL for 2,000 Hz tone and 80 dB SPL for 100 Hz tone. Auditory stimuli were presented using GENELEC 6010A active loudspeaker placed at 0° in front of the participant and visual stimuli were presented on an LG 23MB35PM monitor at a refresh rate of 60 Hz with a resolution of 1,920 × 1,080 in a distance of 1*m*.

### 2.3 Implicit association test and procedure

We explored the implicit associations of two audiovisual cross-modal correspondences: size CMC (visual size and auditory pitch) and elevation CMC (visual elevation and auditory pitch) were explored using a modified Implicit Association Test (Parise and Spence, 2012) similarly to Bolam et al. (2022). Size CMC represents a statistical type of correspondence, linking high-pitched sounds to smaller objects and low-pitched sounds to larger objects, which might stem from associations grounded in frequent co-occurrences of given stimuli (Spence, 2011; Parise and Spence, 2012; Gallace and Spence, 2006). Elevation CMC represents a semantic type of correspondence, which manifests from symbolic relationships between sensory inputs, linking high pitch and high elevation, low pitch, and low elevation (Spence, 2011; McCormick et al., 2018; Zeljko et al., 2019).

**Figure 1:**
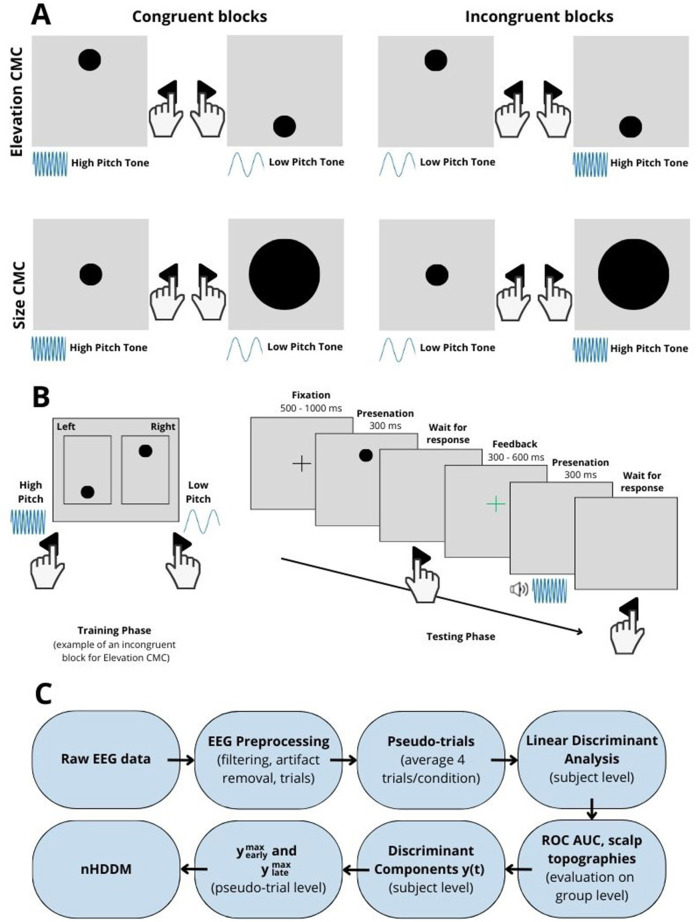
**(A)** Example of response key-stimulus mappings for the implicit association test. The two different types of audiovisual cross-modal correspondences (CMCs)—elevation (top), and size (bottom)—were introduced as a between-subject effect. To prevent biased associations between keys and stimuli features (e.g., right key and high pitch) the mappings were balanced in regards to the response keys. **(B)** In each block of trials, participants first memorized the response key-stimulus mapping during a training phase. By pressing the respective response buttons, they played the auditory stimuli assigned to each key, allowing them to learn the mappings. In the subsequent testing phase, they were presented with a single stimulus per trial (auditory or visual) and instructed to categorize the stimulus by pressing the correct response key, as assigned in the corresponding block. The congruency and the association between response buttons and stimulus features were systematically manipulated across blocks. **(C)** Overview of the methodology. First, raw EEG data were preprocessed. Then, pseudo-trials were created by averaging four trials of the same condition (congruency, modality, answer: correct/incorrect) enabling reliable neural signatures. Linear Discriminant Analysis (LDA) was applied to each participant's data to classify conditions and to obtain time-resolved classifier performance (ROC AUC), discriminant components *y*(*t*), and scalp topographies. From the LDA results, the maximum amplitudes $y_{early}^{max}$ and $y_{late}^{max}$ for early and late windows were first extracted for each pseudo-trial. Finally, these amplitudes were used as neural regressors in a neurally informed hierarchical drift-diffusion model (nHDDM) to assess the impact of neural activity on decision-making processes. Given the hierarchical nature of the model, group-level and participant-level parameters are estimated jointly.

The two different types of CMCs were introduced as a between-subject effect. The experiment involved four distinct stimuli: two auditory (high and low pitch) and two visual (small and large circles for size CMC; high and low elevation for elevation CMC). Each experimental block consisted of a training and testing phase. During the training phase, participants practiced a stimulus-response mapping with two response keys. One auditory and one visual stimulus were mapped to one response key, and the remaining stimuli were mapped to the other (Figures 1A, B). By pressing the respective response buttons, they played the auditory stimuli assigned to each key, allowing them to learn the mappings. There was no time limit to practice the mapping. This mapping could be either congruent (i.e., high pitch with high elevation or small circle) or incongruent (i.e., high pitch with low elevation or big circle) and was balanced across blocks. To prevent biased associations between keys and stimuli features (e.g., right key and high pitch) the mappings were also balanced in regards to the response keys and altered in each experimental block resulting in four key-stimulus mappings per CMC type (Figure 1A). In the testing phase, participants classified one stimulus at a time using the designated button, striving for speed and accuracy, with immediate feedback provided via color-coded fixation cross (Figure 1B). The correct answer was when the appropriate key assigned to the stimuli for the given condition was pressed. For example in Figure 1B, if the left key was pressed for the first trial (high visual elevation, mapped to the right key), the answer would be incorrect and the red fixation cross would be displayed.

### 2.4 Procedure

The participants were seated 1*m* from the monitor and loudspeaker in an acoustically optimized booth. The experiment procedure started with instructions to respond to stimulus presentation as fast and as accurately as possible by pressing the left or right response button. Each trial began with a presentation of a fixation cross at the center of the screen for a randomized period (uniform distribution 500 to 1,000 ms). Next, one of the four stimuli, visual or auditory, was presented for 300 ms. After the participant's response feedback in the form of a red (incorrect answer) or green (correct answer) cross was provided for a randomized period (uniform distribution 300 to 600 ms). The experiment in total comprised 1,280 trials in 16 blocks (i.e., 80 trials per block, 20 per stimulus feature, resulting in 640 congruent and incongruent trials, 320 congruent and incongruent trials per stimulus modality) and lasted approximately 3 h, including 1.5 h for EEG setup and 1.5 h for the experiment. The methodology for processing EEG data is illustrated in Figure 1C.

### 2.5 Behavioral data analysis

Response times and accuracy were recorded for each participant as dependent measures of behavioral performance. RTs shorter than 250 ms and longer than 3·1.4826·*MAD* (median absolute deviations) were rejected as outliers (Leys et al., 2013). That led to the rejection of 7.2% of all trials, leaving 35,228 trials to analyze. RT and accuracy data were not normally distributed, therefore median participant RTs and mean accuracy were computed and subjected to paired random permutation tests. Effect sizes were estimated using Cliff's Delta for RT data (non-parametric measure of effect size) and Cohen's *d* for accuracy data.

### 2.6 EEG data preprocessing

EEG data were recorded for each participant using 64 channels with an actiChamp Plus^TM^ (Brain Products GmbH) amplifier system at a sampling rate of 2,500 Hz and an analog anti-aliasing filter with an upper cutoff frequency of 690 Hz. Recorded data were preprocessed in MATLAB R2023a using the EEGLAB Toolbox (v. 2023.1) (Delorme and Makeig, 2004). First, data were filtered using a linear-phase high-pass filter with a 1 Hz cutoff frequency. Then, the EEG data were down-sampled to 200 Hz. Next, data were re-referenced to average reference, and Artifact Subspace Reconstruction was used to identify noisy channels. These channels were removed after visual inspection and reconstructed using spherical spline interpolation. Potential signal artifacts were removed using independent component analysis; components identified by ICLabel (Pion-Tonachini et al., 2019) as being eye- or muscle-related and components related to noisy electrode channels were removed after visual inspection. EEG data from three participants were excluded from further analysis due to excessive noise or poor signal quality.

Subsequent processing of EEG data was conducted via custom scripts in Python (v. 3.11) using the MNE library (v. 1.6.0) (Larson et al., 2024; Gramfort et al., 2013) and the scikit-learn library (v. 1.4.1) (Pedregosa et al., 2011). For further analysis, the data were additionally low-pass filtered with a low cutoff frequency at 30 Hz. Epochs between –0.3 to 0.7 s relative to stimulus onset were created, with baseline correction using the signal from –0.3 to 0 s relative to stimulus onset.

### 2.7 Single trial EEG data analysis

To quantify whether EEG activity differs between experimental conditions, a linear multivariate single-trial analysis based on regularized linear discriminant analysis (LDA) was performed (Li et al., 2024; Franzen et al., 2020; Kayser et al., 2017; Blankertz et al., 2011; Philiastides et al., 2006, 2014; Sajda et al., 2009; Parra et al., 2005, 2002). LDA is a technique used for dimensionality reduction and data classification, which integrates information across the multidimensional electrode space, instead of across trials as in trial-average event-related method methods. LDA with sliding window approach was used to learn the spatial weighting matrix *w* (spatial filter) which was then applied to generate the one-dimensional projection *y*(*t*) of the multichannel EEG signal that maximally discriminates between given two conditions of interest within each time window *t* and for each trial *i*


(1) $$\begin{array}{l} y\left(t\right)=w^{T}x\left(t\right)=\overset{D}{\underset{i=1}{\sum }}w_{i}x_{i}\left(t\right) \end{array}$$

Here, *T* denotes the transpose operator, and *D* refers to the number of EEG channels. The epoched EEG data *x*_*i*_(*t*) were segmented into a sliding window of 60 ms shifted by 5 ms increments. For each time window, the EEG data were averaged across time. Pseudo-trials were created by averaging four trials from a single condition—congruency, modality, and correct/incorrect (Scrivener et al., 2023). More extreme discriminant component amplitude *y*(*t*) values indicate a higher likelihood of categorizing the trial as one of the conditions, while values near zero suggest less discriminative component amplitudes. Specifically, in our case, higher negative amplitudes indicate more evidence for incongruent stimuli, while higher positive amplitudes indicate more evidence for congruent stimuli. The classifier performance was obtained for each participant as the receiver operating characteristic area under the curve (AUC), gained by a 20-fold Monte-Carlo stratified cross-validation procedure. Both the *y*(*t*) and the AUC were aligned with the onset of the sliding window.

Group significance thresholds for the discriminator performance, rather than assuming an AUC of 0.5 as chance performance, were determined using bootstrap analysis. In this analysis, congruent and incongruent labels were randomized and subjected to a separate 20-fold Monte Carlo stratified cross-validation procedure. This randomization process was repeated 1,000 times. For each randomization, we computed the group-averaged AUC score value and identified the maximal AUC score value over time, building a distribution of AUC score values. From this distribution, we extracted the 99th percentile, which, due to the maximum operation, provides a Family-Wise Error Rate of *p* = 0.01, corrected for multiple comparisons over time points (Holmes et al., 1996; Nichols and Holmes, 2002).

Scalp topographies corresponding to the given classifier were determined by estimating the forward model, defined as the normalized correlation between the discriminant component and the EEG activity (Parra et al., 2005). To identify components that reflect physiologically distinct processes, we applied k-means clustering with a Euclidean distance metric (Duda et al., 2012) to the forward models in the time window of significant classification performance. We optimized the number of clusters (representing different time windows with similar scalp topographies) using silhouette values similarly to Blank et al. (2013) and Franzen et al. (2020). Our results remained robust regardless of the choice of criterion.

Stimulus and response-locked trials were classified to identify early sensory and late decisional processes sensitive to congruency. For the stimulus-locked trials, classification was also performed to determine different stimuli features (high-/low-pitch for auditory trials, high-/low-elevation, or small-/big-size circle for visual trials), so as to better understand the timing of sensory-related processes.

### 2.8 Neurally informed hierarchical drift-diffusion modeling

The Hierarchical Drift-Diffusion Model (HDDM) is a computational framework used to model and analyze decision-making processes. It builds upon the traditional Drift-Diffusion Model (DDM), which characterizes decision-making as a process where noisy evidence accumulates over time until it reaches one of two decision boundaries, representing different choice alternatives (e.g., correct vs. incorrect choices). The key parameters of the DDM include the drift rate, representing the average rate of evidence accumulation; the decision boundary, which reflects the amount of evidence required to make a decision; the starting point, indicating any bias toward one of the decision boundaries; and the non-decision time, accounting for processes such as sensory encoding and motor response latency (Ratcliff and McKoon, 2008; Ratcliff, 1978; Forstmann et al., 2016).

The HDDM extends this framework by incorporating a hierarchical Bayesian modeling approach, allowing for the simultaneous estimation of group-level and individual-level parameters. This hierarchical structure leverages the assumption that individual participant data are random samples from a broader population distribution, thereby improving parameter estimation accuracy, particularly in datasets with a low number of trials (Wiecki et al., 2013; Ratcliff and Childers, 2015; Vandekerckhove et al., 2011). The model fitting in HDDM is executed through Markov Chain Monte Carlo (MCMC) sampling, which iteratively adjusts prior distributions of estimated parameters using a likelihood function that maximizes the probability of the observed data (Gamerman and Lopes, 2006). An advantage of the HDDM is its ability to incorporate external variables, such as neural data (e.g., EEG and fMRI signals), as regressors to inform the estimation of specific decision-making parameters. This feature allows for a more direct examination of how neural activity influences parameters like drift rate or decision boundary (Delis et al., 2022; Frank et al., 2015; Franzen et al., 2020). The use of the Bayesian hierarchical frameworks in HDDM also enables the estimation of posterior distributions for each parameter, thereby quantifying the uncertainty associated with these estimates (Navarro and Fuss, 2009; Gelman, 2003).

#### 2.8.1 Fitting

The HDDM implementation involves an “accuracy-coding” approach, where the model is fitted to RT distributions assuming that the upper and lower decision boundaries correspond to correct and incorrect choices, respectively. For each decisional process, the HDDM provides estimates for key parameters such as drift rate δ, decision boundary θ, and non-decision time τ, while the starting point *z* was fixed at the midpoint between the decision boundaries in cases without a priori bias (Philiastides et al., 2014; Wiecki et al., 2013).

To fit the HDDM, the HDDM Python library (Wiecki et al., 2013) was used, which provides a suite of tools for hierarchical Bayesian modeling of decision-making processes. The model fitting was executed using a Docker container, designed to streamline the HDDM workflow (Pan et al., 2022). When fitting the model, special attention was given to the convergence and stability of the MCMC chains. Four chains were run with 11,000 samples, with the initial 5,000 “burn-in” samples discarded and thinned by factor 20 to reduce auto-correlation. Convergence was assessed using the Gelman-Rubin $\hat{R}$ statistic, with values between 0.98 and 1.02 indicating reliable convergence across chains (Gelman and Rubin, 1992). For model comparison, the Deviance Information Criterion (DIC) was used, a metric widely applied in the assessment and comparison of hierarchical models (Spiegelhalter et al., 2002). DIC selects the model that best balances goodness-of-fit with model complexity, where lower DIC values indicate models that achieve a better trade-off between high likelihood and minimal degrees of freedom.

#### 2.8.2 EEG regressors

To inform the fitting of the HDDM to behavioral data, we incorporated the results from EEG discriminant analysis (Figure 1C). Specifically, EEG discriminant component amplitudes from pseudo-trials (see Section 2.7) were used as regressors for drift rate, decision boundary, and non-decision time to assess their linear relationship with these decision-making parameters. The RT for each pseudo-trial was obtained by computing an average. Based on our observations, where lower RTs were consistently found for congruent trials, we hypothesized that the component amplitudes in congruent trials would predict increases in drift rate, decreases in decision boundary, and shorter non-decisional processes. Conversely, for incongruent trials, we expected the component amplitudes to predict decreases in drift rate, increases in decision boundary, and longer non-decisional processes. We also included stimulus modality (*S*) as a predictor for drift rate. During the model fitting within the HDDM framework, we constructed regressors using the EEG discriminant amplitudes for congruent and incongruent trials, separately for different types of CMC (size-pitch and elevation-pitch), as follows:


(2) $$\begin{array}{l} \delta =\alpha_{0}+\alpha_{1}*y_{early}^{max}+\alpha_{2}*y_{late}^{max}+\alpha_{3}*S \end{array}$$

(3) $$\begin{array}{l} \theta =\beta_{0}+\beta_{1}*y_{early}^{max}+\beta_{2}*y_{late}^{max} \end{array}$$

(4) $$\begin{array}{l} \tau =\gamma_{0}+\gamma_{1}*y_{early}^{max}+\gamma_{2}*y_{late}^{max} \end{array}$$

Here, $y_{early}^{max}$ and $y_{late}^{max}$ represent the maximum discriminator amplitudes of subject-specific, stimulus-locked EEG components that capture the highest classification performance between congruent and incongruent trials. Early EEG components were derived from individual peak AUC score values and corresponding forward models in the time range of 125–350 ms post-stimulus for Size CMC and 125–300 ms for Elevation CMC. Late EEG components were based on individual peak AUC scores and corresponding forward models in the time range of 450–600 ms for both CMC types (see Section 3.2). The coefficients α_1_, β_1_, γ_1_, and α_2_, β_2_, γ_2_ weight the slope of each parameter according to the values of $y_{early}^{max}$ and $y_{late}^{max}$, respectively, with intercepts α_0_, β_0_, and γ_0_, on a trial-by-trial basis for each subject, congruency condition, and type of CMC. *S* denotes the stimulus modality (either visual or auditory). Note that $y_{early}^{max}$ and $y_{late}^{max}$ were normalized for the effects of congruency (ignoring the sign), so that higher amplitude signifies more sensory evidence.

#### 2.8.3 Hypothesis testing

Our statistical approach relies on Bayesian hypothesis testing rather than a classical frequentist approach. Specifically, to test our hypotheses regarding the HDDM results, we employed a form of posterior log-odds testing. This approach allows us to assess the strength of evidence supporting our predefined hypotheses using posterior distributions. Specifically, we employed the built-in functions of the HDDM toolbox (Wiecki et al., 2013) to compute the posterior distributions of the regression coefficients. Our hypotheses predicted decreases in reaction times (RTs) for congruent trials compared to incongruent trials, as well as in congruent trials decreases in RTs for incorrect responses compared to correct responses. For congruent drift rate (δ^*C*^), decision boundary (θ^*C*^), and incongruent non-decision time (τ^*I*^) regression coefficients, posterior probability densities were calculated based on the proportion of posterior samples greater than zero (*P*(δ^*C*^ > 0); [*P*(θ^*I*^ > 0); *P*(τ^*I*^ > 0)]. For incongruent drift rate (δ^*I*^), decision boundary (θ^*I*^) and congruent non-decision time (τ^*C*^) regression coefficients, posterior densities were calculated from the proportion of posterior samples less than zero (*P*(δ^*I*^ < 0); [*P*(θ^*C*^ < 0); *P*(τ^*C*^ < 0)].

To quantify the evidence for each hypothesis, we applied the logit transformation to the proportions, resulting in posterior log-odds for each coefficient (Ince et al., 2021). Given the hierarchical nature of our model, where group-level and participant-level parameters are jointly estimated, this Bayesian approach is particularly suited as it avoids the independence assumptions inherent in traditional frequentist null-hypothesis significance testing (Wiecki et al., 2013). To further assess the predictive strength of the regression coefficients, we calculated the posterior log-odds for a hypothetical sample with a false-positive rate of α = 0.05 (i.e., a 95% true-positive threshold, 2.994 log-odds). Regression coefficients with log-odds proportions greater than the threshold provide evidence for a non-zero effect on posterior parameter estimates, thus supporting our hypotheses. While high log-odds indicate confidence in the direction of the effect, they do not directly quantify the strength or magnitude of the effect.

## 3 Results

### 3.1 Behavior

**Figure 2:**
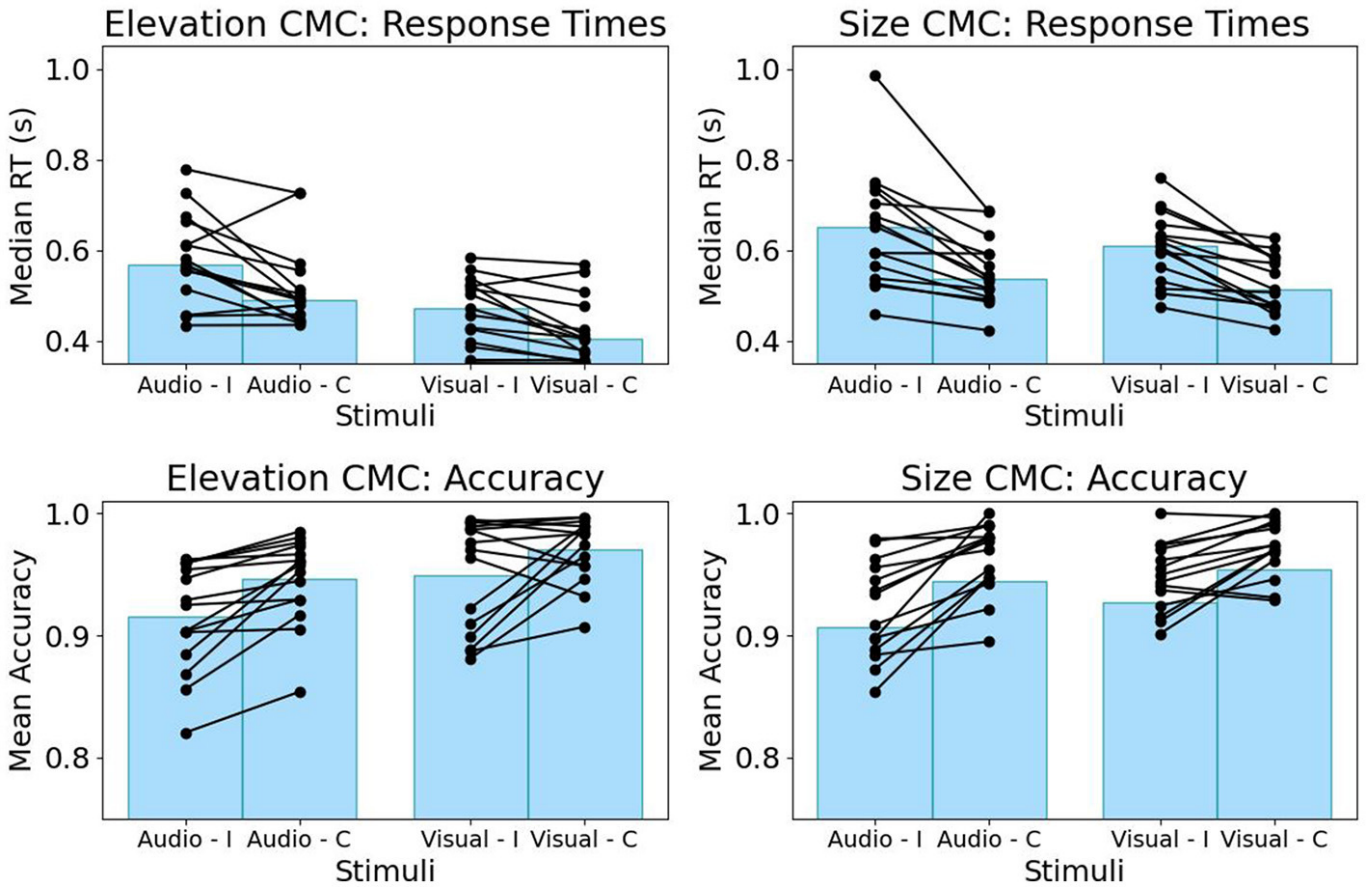
Behavioral task performance for the CMC between elevation and pitch **(left)** and between size and pitch **(right)**. RTs **(top)** and accuracy **(bottom)** are grouped by stimulus modality. Incongruent (I) trials demonstrate higher RTs and lower accuracy compared to congruent trials (C) for both CMCs. Each line represents a participant.

We found a significant difference in RTs and accuracy between congruent and incongruent trials for both types of CMC, where incongruent trials show slower response times and lower accuracy (c.f., Figure 2). This was confirmed by paired random permutation tests for both median RTs (congruent vs. incongruent size CMC, *n* = 15 participants, *p* < 0.001, Cliff's Delta 0.49, median congruent 0.51 s, median incongruent 0.62 s; congruent vs. incongruent elevation CMC, *n* = 15 participants, *p* = 0.004, Cliff's Delta 0.38, median congruent 0.44 s, median incongruent 0.53 s) and mean accuracy (congruent vs. incongruent size CMC, *n* = 15 participants, *p* = 0.012, Cohen's *d* = 0.34, mean congruent 0.94, mean incongruent 0.92; congruent vs. incongruent elevation CMC, *n* = 15 participants, *p* = 0.002, Cohen's *d* = 0.78, mean congruent 0.96, mean incongruent 0.93). The effect size as quantified by Cliff's delta is bigger for differences in response times for size-pitch correspondence, whereas, for accuracy, the bigger effect as quantified by Cohen's *d* can be observed for correspondence between visual elevation and auditory pitch.

### 3.2 EEG analysis

EEG data were analyzed to identify neurophysiological processes sensitive to congruency, regardless of modality. For each participant, a multivariate linear discriminant analysis was conducted to estimate spatial weights that maximally discriminated congruent from incongruent trials in sliding windows of 60 ms. Applying these weights to the pseudo-trial EEG signals resulted in a projection *y*(*t*) of the multichannel signal that maximally distinguishes between the two congruency conditions. The amplitude of *y*(*t*) serves as an indicator of neural evidence, with higher amplitudes suggesting greater evidence for one of the conditions. Specifically, in our case, higher negative amplitudes indicate more evidence for incongruent stimuli, while higher positive amplitudes indicate more evidence for congruent stimuli.

**Figure 3:**
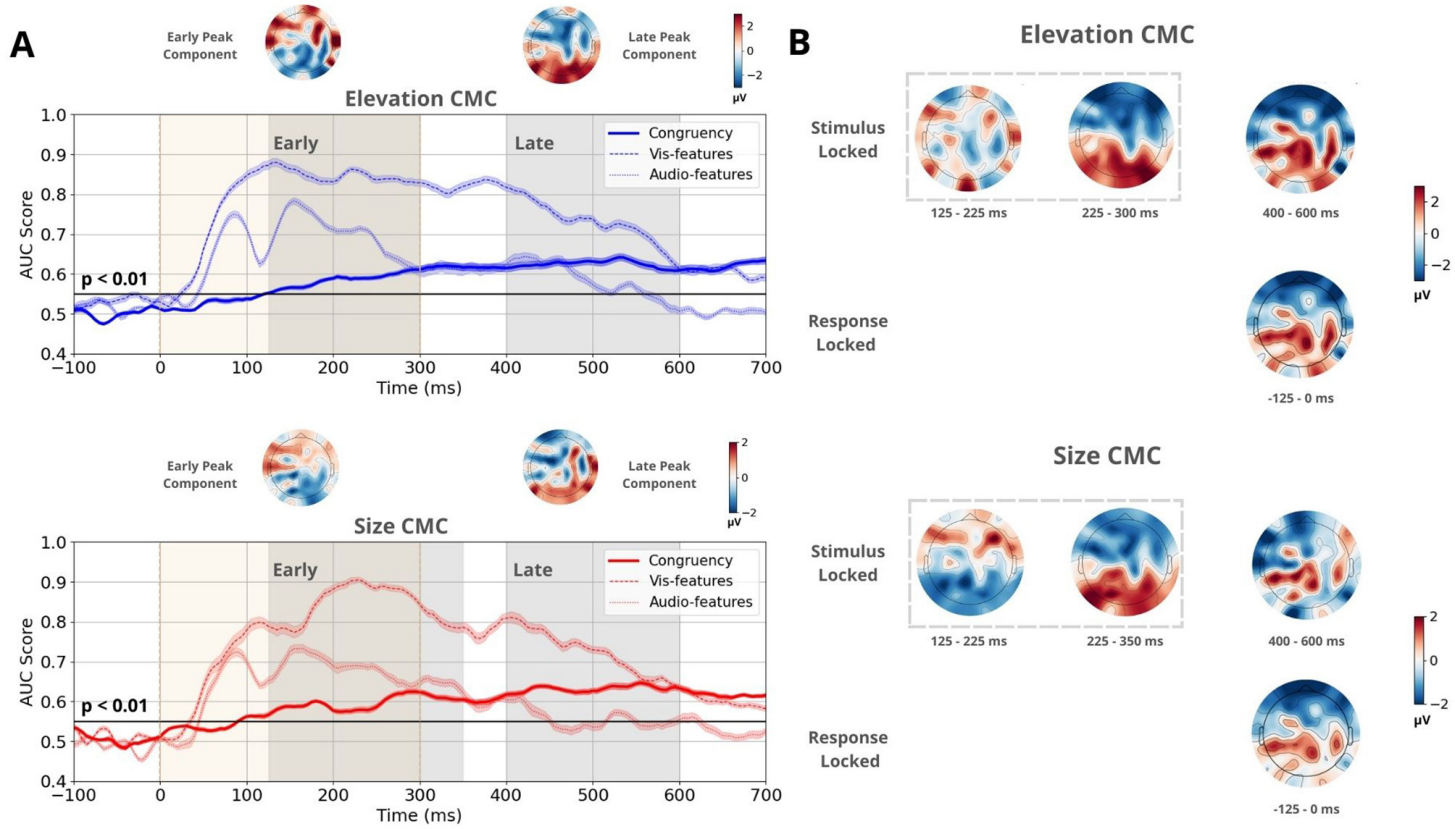
Multivariate linear discriminant analysis results for the CMC between elevation and pitch and between size and pitch. **(A)** Time course of the mean classifier performance of stimulus-locked trials, quantified by the AUC score obtained through a 20-fold Monte Carlo stratified cross-validation procedure. The bold line represents performance based on congruency (congruent vs. incongruent), while the dotted and dashed lines represent performance based on stimulus features (auditory high-low pitch and visual high-low elevation/small-big circle, respectively). The black line indicates the group permutation threshold at *p* < 0.01. Beige vertical bars indicate stimulus presentation. Shaded gray vertical bars denote the Early and Late EEG component windows, which were determined using temporal clustering of the topographies of the associated forward models. Mean forward model topographies representing Early and Late peak components for both CMCs. These topographies were computed by averaging the topographies at the time windows corresponding to maximum performance during the Early and Late time windows for each participant. **(B)** Forward model topographies of stimulus and response-locked trials sensitive to congruency at time windows determined by temporal clustering. Two spatiotemporal patterns were observed during the Early component. During the Late component, one spatiotemporal pattern was present, which is identical to the activation pattern in response-locked trials.

The discrimination performance for stimulus-locked congruent-vs-incongruent trials exceeded the chance level at 90 ms for size CMC and at 125 ms for elevation CMC (see Figure 3A). Within the time range of significant classifier performance, we applied temporal clustering on the mean forward model topographies to identify the number of relevant components. Scalp topographies were estimated using a forward model (Parra et al., 2005), which identified three distinct spatiotemporal patterns across both CMCs (Figure 3B). Two of these patterns emerged early, mostly during stimulus presentation (first 300 ms). The first pattern was observed between 125–225 ms, the second between 230–300 ms and 230–350 ms for the elevation and size CMCs, respectively. The latter component was consistent across both CMCs and exhibited a pronounced front-back polarization. The third spatiotemporal pattern, present between 400–600 ms, showed a prominent centroparietal activation cluster for both CMCs. The transition point between the earlier components and the third component occurred around 380 ms for size CMC and around 350 ms for elevation CMC, with a longer transition period observed for elevation CMC.

The congruent-vs.-incongruent discriminant analysis was also performed on response-locked trials. Temporal clustering of the for topographies within the time range of significant discrimination identified one spatiotemporal pattern between –125–0 ms relative to stimulus onset consistent across both CMCs. It showed a centroparietal activation cluster identical to the third pattern in stimulus-locked trials (Figure 3B).

Similarly, we applied the same discriminant analysis to identify components that distinguish between stimulus features (Figure 3A). This analysis was performed separately for auditory and visual trials. For elevation CMC, low- and high-pitched tones and low- and high-elevation circles were classified. For size CMC, low- and high-pitched tones and small- and large-sized circles were classified. Discriminator performance rose above the chance level as early as 40 ms for visual stimuli and 50 ms for auditory stimuli. Notably, the two early congruency-discriminating spatiotemporal patterns temporally coincided with high classification performance of stimulus features. Based on these observations, the Early component was defined between 125–300 ms for size CMC and between 125–350 ms for elevation CMC, and was attributed a sensory-related role. The Late, post-sensory component was defined between 400–600 ms for both CMCs.

**Figure 4:**
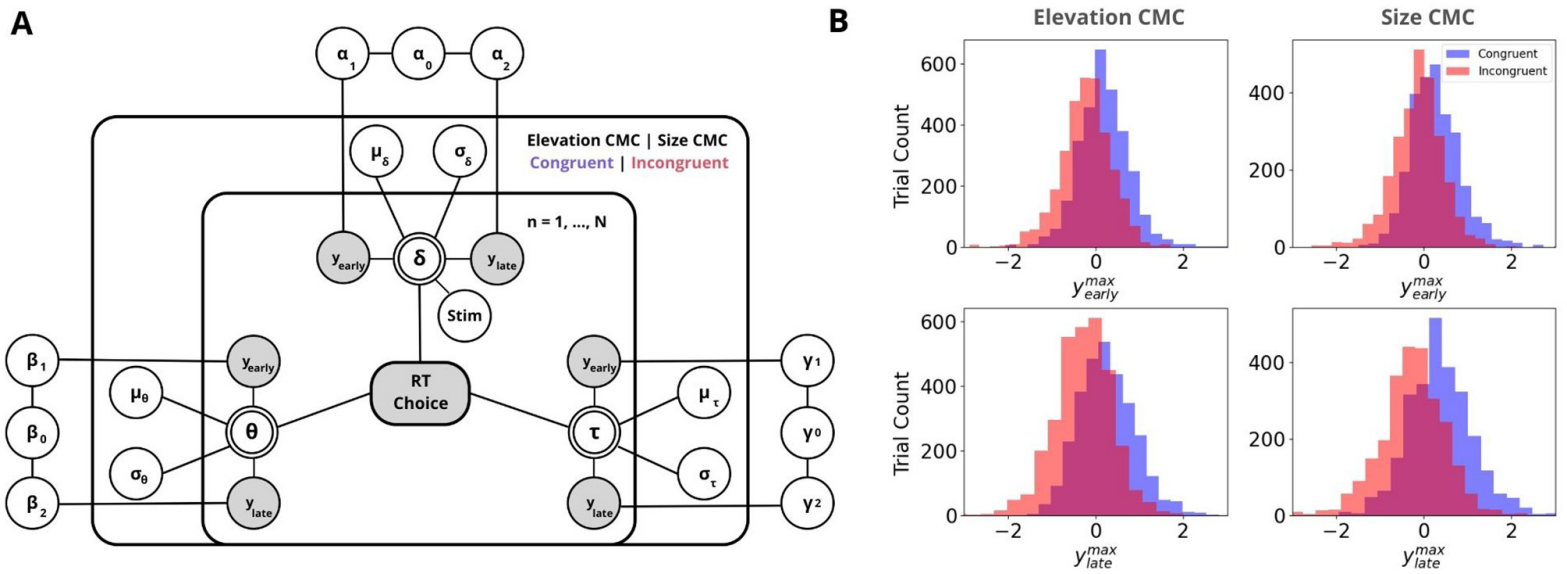
Neurally-informed cognitive modeling. **(A)** Graphical representation of the Bayesian hierarchical framework used to estimate neurally-informed HDDM parameters. Circular nodes represent continuous random variables, with shaded nodes indicating observed or computed data, such as behavioral data (RTs and Choices) and EEG component discriminator amplitudes *y*'s. Double-bordered nodes represent deterministic variables, which are defined based on other variables. Plates indicate a hierarchical structure for modeling multiple random variables, where the inner plate represents participants (*n* = 1,... , N), the outer plate represents CMC types and congruency conditions (Elevation CMC: pitch and elevation; Size CMC: pitch and visual size, Congruent|Incongruent). Parameters are modeled as random variables with inferred means μ and variances σ^2^, constrained by estimates inferred across CMC conditions. External plates depict the constructed regression coefficients, which serve as predictors for drift rate δ, decision boundary θ, and non-decision time τ. **(B)** Discriminator amplitudes *y* for Congruent (blue) and Incongruent (red) components are shown as histograms, separately for the Early (top) and Late (bottom) EEG components for both CMC types. Negative values reflect neural evidence for incongruency, while positive values reflect evidence for congruency.

Additionally, maximal discriminator performance for congruency was determined during the Early and Late time windows for each participant. At the corresponding times, projection amplitudes $y_{early}^{max}$ and $y_{late}^{max}$ were collected (Figure 4B). On average, peak performance during the Early time window occurred at 241 ms for elevation CMC and 253 ms for size CMC. During the Late time window, the mean peak performance was observed at 514 ms for elevation CMC and 503 ms for size CMC. The corresponding mean peak scalp topographies for the Early and Late time components showcase opposite polarities and are consistent across both CMCs (Figure 3A upper topographies).

### 3.3 Neurally informed cognitive modeling

Following the identification of neural signatures that differentiate congruent from incongruent trials, we aimed to explore how trial-to-trial neural variations contribute to perceptual decision-making across different types of CMCs. To achieve this, we employed a neurally-informed variant of the Hierarchical Drift Diffusion Model (Wiecki et al., 2013, Figure 4A). By incorporating EEG discriminant component amplitudes, $y_{early}^{max}$ and $y_{late}^{max}$, as regressors for HDDM parameters, we constrained the model with neural data, allowing us to assess the impact of neural activity on perceptual decision-making processes between congruent and incongruent trials.

**Figure 5:**
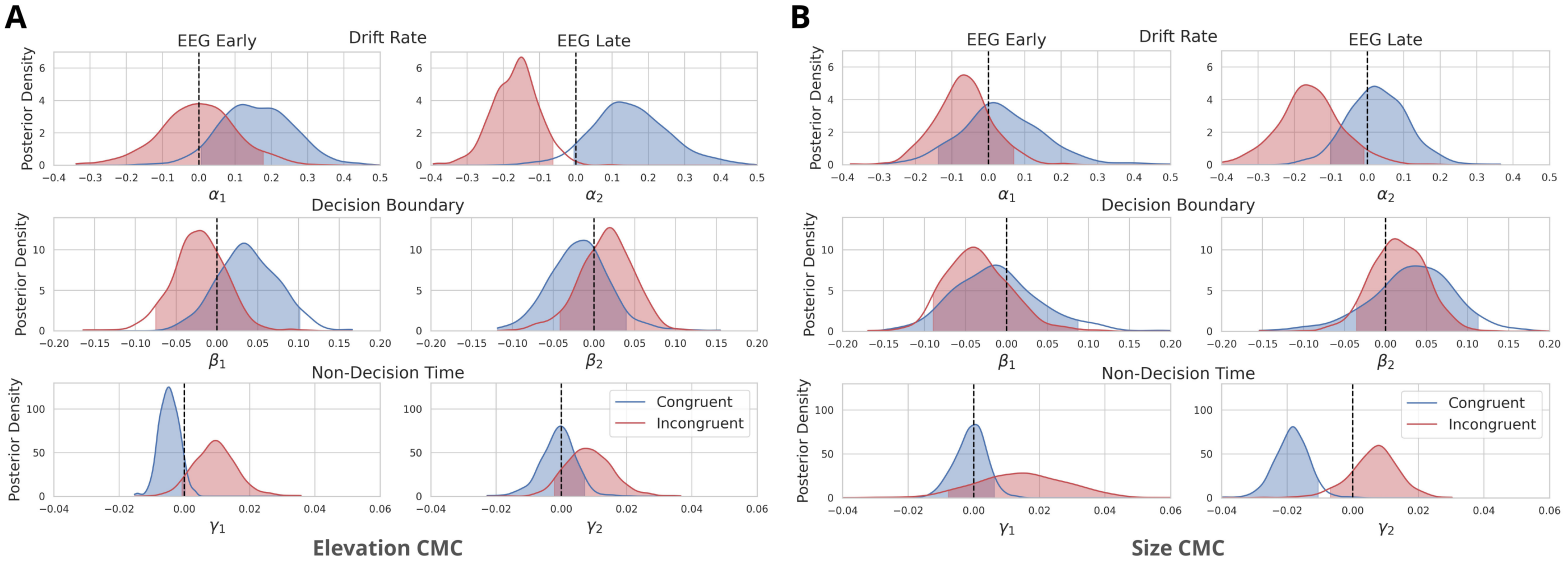
Neurally-informed cognitive modeling results for two types of cross-modal associations: **(A)** between elevation and pitch-elevation CMC and **(B)** between size and pitch-size CMC. The figure depicts posterior density distributions of the estimated regression coefficients for drift rate (α's), decision boundary (β's), and non-decision time (γ's). These coefficients are linked to Early and Late EEG component discriminator amplitudes *y*'s for both congruent and incongruent trials. The regression coefficients were estimated using a neurally-informed Hierarchical Drift Diffusion Model, based on data from *n* = 27 independent participants and 11,432 trials. Shaded areas represent the 95% probability mass, while dashed lines indicate the zero point.

In brief, the HDDM decomposes task performance into three main components: drift rate (δ) for evidence accumulation, decision boundary (α) for the amount of evidence required to make a decision, and non-decision time (τ) for processes such as stimulus encoding and motor response. To examine the relationship between neural activity and these parameters, we used EEG-derived regressors–$y_{early}^{max}$ and $y_{late}^{max}$–normalized for congruency and included them as predictors in the HDDM for drift rate, decision boundary, and non-decision time. The estimated regression coefficients (α_1_, β_1_, γ_1_ for $y_{early}^{max}$ and α_2_, β_2_, γ_2_ for $y_{late}^{max}$) allowed us to assess how neural activity influenced decision-making (see Figure 4B, Figure 5).

**Figure 6:**
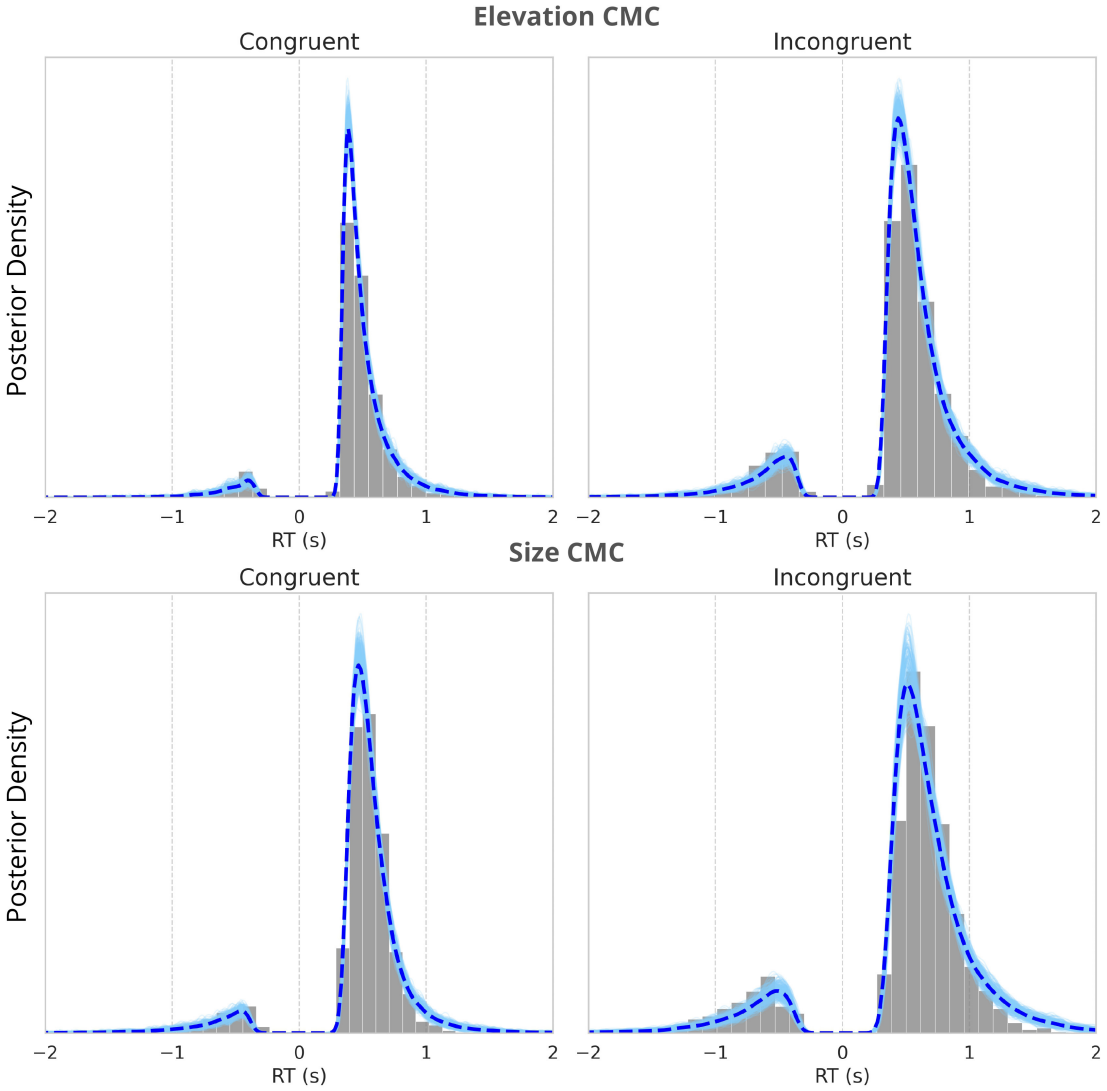
Posterior predictive checks of the neurally-informed HDDM fitting. The histograms of the observed reaction time distributions, with the posterior predictive distributions from the nHDDM model overlaid. The dashed line represents the mean of the posterior predictive distributions. The alignment between the observed data and the model's predictions demonstrates the model's ability to capture the characteristics of reaction times across all conditions. Negative RTs indicate incorrect responses. The figure compares the model fits for congruent trials **(left)** and incongruent trials **(right)** across two types of CMCs–elevation and size **(upper panel)** and size and pitch **(lower panel)**.

These EEG-derived regressors capture early bottom-up and late top-down modulations in neural activity linked to associative congruency, allowing us to investigate how these variations influence perceptual decision formation. The component amplitudes were used to reflect higher discriminant activity between congruent trials and incongruent trials. By comparing the obtained HDDM parameter values between congruent and incongruent trials, as well as across different types of CMCs, we aimed to identify the processes contributing to the observed behavioral differences, such as improved task performance and smaller RTs for congruent trials (as depicted in Figure 2). We found a good model fit with the $\hat{R}$ values between 0.98 and 1.02 for all estimated parameters indicating reliable convergence across chains (for posterior predictive checks see Figure 6).

For elevation CMC, Early EEG components had a significant predictive and positive effect on drift rate in congruent trials, with [$P\left(\alpha_{1}^{C}>0\right)=0.955$, log-odds = 3.053, Figure 5A]. Similarly, Late EEG components positively influenced drift rate in congruent trials [$P\left(\alpha_{2}^{C}>0\right)=0.94$, log-odds = 2.75, Figure 5A], though this effect did not meet the significance threshold (2.994 log-odds). In incongruent trials, Early EEG components did not significantly affect drift rate, with [$(P\left(\alpha_{1}^{I}<0\right)=0.503$, log-odds = 0.011, Figure 5A], showing minimal predictive power. However, for incongruent trials, Late EEG components had a strong significant negative effect on drift rate, with [$P\left(\alpha_{2}^{I}<0\right)=0.997$ log-odds = 5.855, Figure 5A], indicating that late neural activity significantly reduces evidence accumulation in incongruent trials. Visual modality consistently influenced drift rate positively in congruent and incongruent trials, with $P\left(\alpha_{3}^{C}>0\right)=1.0,P\left(\alpha_{3}^{I}>0\right)=1.0$, suggesting a reliable positive effect across both conditions. Early EEG and Late EEG components had no significant effect on decision boundary [$P\left(\beta_{1}^{C}<0\right)=0.149$, log-odds = –1.743, $P\left(\beta_{2}^{C}<0\right)=0.706$, log-odds = 0.877, Figure 5A] for congruent trials. For incongruent trials, early EEG components were also non-significant [$P\left(\beta_{1}^{I}>0\right)=0.243$, log-odds = –1.137], and late EEG components failed to show a significant effect [$P\left(\beta_{2}^{I}>0\right)=0.691$, log-odds = 0.807, Figure 5A].

For size CMC, the effects of Early EEG components on drift rate in congruent trials were not as pronounced, with $P\left(\alpha_{1}^{C}>0\right)=0.632$, log-odds = 0.539, see Figure 5B. Late EEG components also positively influenced drift rate [$P\left(\alpha_{2}^{C}>0\right)=0.639$, log-odds = 0.571, Figure 5B], though again, this effect was weaker and not significant compared to Elevation CMC. In incongruent trials, early EEG components had a non-significant moderate negative effect on drift rate, with $P\left(\alpha_{1}^{I}<0\right)=0.826$ (log-odds = 1.556, Figure 5B), while late EEG components had a significant negative effect [$P\left(\alpha_{2}^{I}<0\right)=0.956$, log-odds = 3.072, Figure 5B], indicating that late neural activity disrupts evidence accumulation in incongruent trials. Visual modality had a strong positive effect for both congruent and incongruent trials, with $P\left(\alpha_{3}^{C}>0\right)=0.966,P\left(\alpha_{3}^{I}>0\right)=0.909$. Early and Late EEG components had no significant effect on decision boundary for congruent trials, with $P\left(\beta_{1}^{C}<0\right)=0.627$ (log-odds = 0.52); $P\left(\beta_{2}^{C}<0\right)=0.227$, log-odds = –1.228, Figure 5B. Also for incongruent trials, both Early and Late EEG components had no significant effect on decision boundary, with $P\left(\beta_{1}^{I}>0\right)=0.189$ (log-odds = -1.459), and $P\left(\beta_{2}^{I}>0\right)=0.690$, log-odds = 0.8, Figure 5B.

In terms of non-decision time, the results differed between Size CMC and Elevation CMC: For Elevation CMC Early EEG components had a strong although not significant effect on non-decision time - positive for congruent trials [$P\left(\gamma_{1}^{C}>0\right)=0.94$, log-odds = 2.75] and negative for incongruent trials [$P\left(\gamma_{1}^{I}<0\right)=0.931$, log-odds = 2.609, Figure 5A]. Late EEG components showed a non-significant positive effect [$P\left(\gamma_{2}^{C}>0\right)=0.557$, log-odds = 0.231] for congruent trials and a moderate negative effect [$P\left(\gamma_{2}^{I}<0\right)=0.891$, log-odds = 2.105, Figure 5A]. For Size CMC, we found a significant negative effect of Late EEG components [$P\left(\gamma_{2}^{C}<0\right)=0.999$, log-odds = 6.526, Figure 5B] non-decision time for congruent trials. Early EEG components had no significant effect on the non-decision time for congruent trials [$P\left(\gamma_{1}^{C}<0\right)=0.55$, log-odds = 0.199, Figure 5B]. For incongruent trials, Early and Late EEG components also showed no significant moderate positive effect on non-decision time [$P\left(\gamma_{1}^{I}>0\right)=0.851$, log-odds = 1.746; $P\left(\gamma_{2}^{I}>0\right)=0.826$, log-odds = 1.556, Figure 5B].

In summary, the analysis revealed that late EEG components significantly reduced the drift rate in incongruent trials across both CMC types. For elevation CMC, early EEG components had a significant positive effect on the drift rate in congruent trials, while early EEG components did not significantly affect the drift rate in incongruent trials. Non-decision time was more prominently affected in the size CMC, where late EEG components had a significant negative impact in congruent trials. Decision boundary parameters remained unaffected by both early and late EEG components across congruent and incongruent trials for both CMC types.

## 4 Discussion

We employed the Implicit Association Test (IAT; Parise and Spence, 2012) combined with concurrent EEG measurements to investigate the neural origins of statistical and semantic types of audiovisual cross-modal correspondences. Our study expands on previous research by examining both auditory and visual modalities to explore whether the effects of congruency generalize across different sensory modalities and CMC types. The behavioral data clearly demonstrated a cognitive cost associated with incongruency, reflected in slower reaction times and reduced accuracy for both statistical CMCs, see Figure 2. These findings underscore the cognitive advantage of congruent associations, reaffirming our assumption that associative congruency shapes decision formation in multisensory contexts (Bien et al., 2012; Zeljko et al., 2019; Franzen et al., 2020; Bolam et al., 2022).

Our goal was to identify the neurophysiological correlates of CMCs and to determine which of those influence perceptual decisions. By applying multivariate linear discriminant analysis and neurally-informed hierarchical drift-diffusion modeling, we were able to identify two distinct functional stages, Early and Late, associated with congruency across both CMC types. Crucially, the behavioral benefits of congruent associations, such as faster RTs and higher accuracy, were linked to these neural correlates. The slower RTs observed in incongruent trials were associated with reduced evidence accumulation (drift rate), which was modulated by the Late EEG component consistently for both CMC types. This suggests that late, post-sensory processing stages play a critical role in modulating perceptual decisions in incongruent trials. Additionally, the consistent positive influence of the visual modality on drift rate, regardless of congruency, underscores the role of sensory integration in audiovisual CMCs. Interestingly, we also found differences between elevation and size CMCs in how drift rate and non-decision time were associated with Early and Late EEG components. This may suggest that the strength or nature of cross-modal associations varies depending on the specific CMC type, potentially reflecting differences in the relative contributions of statistical and semantic processes.

### 4.1 Behavior

Supporting previous research on CMCs, our behavioral data demonstrate the existence of cross-modal correspondences (CMCs) between auditory pitch and visual size (Bien et al., 2012; Gallace and Spence, 2006; Deroy et al., 2013; Spence and Parise, 2012; Parise and Spence, 2012; Sciortino and Kayser, 2023), as well as between auditory pitch and visual elevation (McCormick et al., 2018; Spence, 2011). Using the IAT, we replicated findings from Bolam et al. (2022) and Parise and Spence (2012), who reported faster reaction times (RTs) and higher accuracy for congruent trials in size CMCs (Parise and Spence, 2012). Additionally, we demonstrated that the IAT is also suitable for examining cross-modal associations between auditory pitch and visual elevation. Our participant cohort included individuals from diverse linguistic backgrounds. While linguistic variability could theoretically influence semantic processing, the association between high elevation and high spatial location is likely universal, as it is rooted in shared perceptual and environmental experiences. Consistent mappings between pitch and elevation have been observed across cultures, though subtle variations may arise due to linguistic or cultural influences (Eitan and Timmers, 2010). Future research could further explore how linguistic diversity shapes the processing of semantically mediated cross-modal correspondences. Finally, it is worth noting that the associative congruency observed in our study is relative in nature (Spence, 2019) and does not depend on the absolute pitch frequency or e.g. absolute visual size presented.

### 4.2 Multivariate LDA

Understanding how the brain integrates information from multiple sources during decision-making is crucial for uncovering the mechanisms that allow for efficient and accurate perceptual judgments. The early and late integration hypotheses propose different mechanisms for how multisensory information is combined (Bizley et al., 2016). The early integration hypothesis suggests that sensory inputs from different modalities are combined during initial sensory encoding, supported by evidence of cross-modal interactions in early sensory regions like the primary auditory and visual cortices (Kayser et al., 2008; Foxe and Schroeder, 2005; Rohe and Noppeney, 2015). In contrast, the late integration hypothesis posits that sensory inputs are processed separately and combined later during higher-order decision-making. These two processes may not be mutually exclusive, as recent findings suggest that both early sensory encoding and late decision-related processes contribute to multisensory integration, with early sensory modulations influencing the final decision-making stage (Mercier and Cappe, 2020; Cao et al., 2019; Talsma, 2015; Rohe and Noppeney, 2015, 2016). Our study aimed to investigate how these early (bottom-up) and late (top-down) integration processes manifest in the brain's neural responses to audiovisual cross-modal correspondences.

To test these hypotheses, we conducted a multivariate linear discriminant analysis to identify spatiotemporal patterns sensitive to congruency, revealing distinct early and late neural components. Analysis, similar to the methodology outlined by Bolam et al. (2022) in their study on neurocomputational mechanisms underlying cross-modal associations and their influence on perceptual decisions, was performed. While their study focused on congruency in auditory trials to explore cross-modal correspondence between visual size and auditory pitch, our study aimed to determine activity sensitive to congruency regardless of modality in two types of CMCs. The discrimination performance between congruent and incongruent trials rose above the chance earlier for size CMC, already 90 ms post-stimulus onset, while for elevation CMC the discrimination performance rose above the chance level at 125 ms post-stimulus. However, temporal clustering of the mean scalp topographies gained by the forward model (Parra et al., 2005) revealed consistent neural patterns across both CMCs (Figure 3B).

The Early component was defined within the range of 125–300 ms (extended to 350 ms for the size CMC). Peak performance in this window occurred at approximately 240–250 ms. Our definition of the Early component diverges from that of Bolam et al. (2022), who identified the Early component at 100–110 ms. Whereas they analyzed auditory-only trials, we included both auditory and visual stimuli-locked trials, resulting in components that were sensitive to congruency regardless of modality. Visual trials, in particular, exhibited more sustained discrimination performance for stimulus features compared to auditory trials, which may explain why our congruency discrimination peaked later. Moreover, the congruency discriminator's performance in our study was generally lower and did not precede nor exceed the stimulus-feature discriminators, in contrast to Bolam et al. (2022). This discrepancy between studies is unexpected, however, audiovisual cross-modal associations should be weaker than the clearly perceivable differences between stimulus features, therefore our results appear more plausible. Nonetheless, the relatively early onset of congruency discrimination in our results still suggests a potential interaction between sensory encoding and pre-existing perceptual priors. The overlap between the Early component and the high performance of the stimulus-feature discriminator suggests that audiovisual cross-modal associations may automatically influence early sensory encoding. These associations are likely active early in the perceptual process, shaping neural representations before conscious decision-making takes place. In line with this, our results support the idea that the behavioral benefits of associative congruency, often seen in faster and more accurate decisions, are likely modulated by neural feedback mechanisms that influence the early stages of sensory processing (Sciortino and Kayser, 2023).

The Late component was defined between 400–600 ms and showed a prominent centroparietal activation. This activation pattern is consistent with previously reported late decision-related components (Franzen et al., 2020; Philiastides and Sajda, 2006; Philiastides et al., 2014; Sajda et al., 2009) and resembles the neural signature of decision formation, termed Centro-Parietal Positivity (CPP) (O'Connell and Kelly, 2021; O'Connell et al., 2018; Tagliabue et al., 2019; Herding et al., 2019). This activation showed the same main characteristics as the spatiotemporal pattern we identified in response-related trials (Figure 3B), providing more evidence for the decisional origin of this pattern. In the study by Mercier and Cappe (2020), the authors also linked this late decision-related component, the CPP, to the process of decision formation during multisensory integration. This aligns with our observation of a centroparietal activation cluster during the Late component, indicating that cross-modal congruency modulates decision-related neural processes, likely contributing to the behavioral benefits observed in congruent trials.

The longer transition period between the Early and Late components for elevation CMC (Figure 3A) suggests distinct neural dynamics compared to size CMC. Semantic correspondences, such as pitch-elevation, likely engage higher-order associative regions and involve more abstract cognitive processing, reflected in the slower transition. In contrast, pitch-size correspondences, which are more direct, exhibit a faster transition, indicating reliance on more automatic sensory associations. This extended transition for elevation CMC could indicate greater involvement of top-down processes. Our findings further support the idea that statistical CMCs, such as pitch-size, are more automatic and processed earlier, while semantic CMCs, like pitch-elevation, require more gradual processing and engage top-down mechanisms.

Since individual trials in our study only presented isolated unisensory stimuli, the neural benefits we observed from congruency seem to be driven by perceptual processing rather than purely decision-related mechanisms. Without the confounding effects of simultaneous multisensory stimulation and selective attention, the use of the IAT allowed us to localize the effects of associative congruency to both early sensory-perceptual and late decisional stages.

### 4.3 nHDDM

In this study, we were able to characterize the neural mechanisms underlying the behavioral advantages of cross-modal audiovisual associations. This was achieved through the integration of cognitive modeling with both behavioral and neural data, allowing us to link neural correlates of sensory processing and decision-making to the internal processes driving perceptual decisions. Specifically, the findings highlight that late EEG components significantly reduce the drift rate in incongruent trials across both CMCs. This suggests that late decision-related processes are particularly sensitive to conflicts between sensory modalities. Further, top-down processes may disrupt or slow down evidence accumulation in the presence of incongruent stimuli, likely reflecting the cognitive cost of resolving conflicting information or the need for re-evaluation. Such a role of multisensory incongruence in shaping later sensory processes is also in line with models of multisensory causal inference (Noppeney, 2021; Cao et al., 2019; Rohe and Noppeney, 2015). According to the current working model of multisensory integration, incongruencies between sensory inputs are resolved by determining whether two stimuli are likely to belong to one common origin or to two distinct objects, and the resulting belief in a common cause then shapes whether and how two sensory signals are combined. The underlying neurophysiological processes supposedly reside in higher parietal and frontal brain regions and which emerge later after stimulus onset than the early unisensory processing in low-level regions.

For the elevation CMC, the Early EEG component showed a significant positive effect in congruent trials, supporting the idea that early sensory encoding is more effective when cross-modal stimuli are aligned. Interestingly, the non-decision time was more prominently affected in the size CMC than in the elevation CMC. Late EEG components had a significant negative impact on non-decision time in congruent trials, indicating faster processing when stimuli align, while early EEG components did not show a strong influence. The absence of a significant effect on decision boundary, a parameter that reflects how much evidence is needed before making a decision, suggests that cross-modal congruency primarily influences the rate and timing of sensory evidence accumulation rather than the threshold for making decisions.

Our results show a key difference in the role of the Late component compared to the previous study on CMCs by Bolam et al. (2022). While their study linked the Late component with a decrease in the amount of evidence required to reach a decision (decision boundary), our Late component primarily influenced the drift rate, particularly in incongruent trials. This suggests that in our study, the focus of late neural processing was on evidence accumulation rather than on modulating the decision threshold. Notably, our findings align with those of Franzen et al. (2020), where the Late component shares both the activation pattern and timing and also reflects decision-related processes that modulate the rate of evidence accumulation. Additionally, in Bolam et al. (2022) results, incongruent stimulus-response mappings yielded increased non-decision time estimates, modulated by their Early component (defined around 100-110 ms). This suggests longer stimulus encoding times in incongruent trials. In contrast, our Early component, was found to positively affect the drift rate in congruent trials, emphasizing the role of early sensory encoding when stimuli align across modalities. This might be explained by the fact that our Early component was identified later (125–300 ms), also similarly to Franzen et al. (2020), where they investigated the auditory enhancement of visual object categorization. They found no significant effects of the Early component. As previously mentioned, a notable difference is that (Bolam et al., 2022) only examined auditory trials, while our study incorporated both auditory and visual stimuli, which could explain some of the differences. This modality effect might also indicate that congruency affects sensory processing differently depending on whether stimuli are auditory or visual. The inclusion of modality in our model as a separate factor significantly influenced drift rate in all conditions (congruent and incongruent and both CMCs), but it might be beneficial to examine the interaction between modality and Early or Late EEG components and their effect on model parameters, potentially including these effects also in non-decisional time. Another explanation might be the fact that IAT is a highly decisional task, where one stimulus at a time is presented and therefore the sensory congruency effects on decision-making might be weaker than when presenting both stimuli simultaneously, resulting in the Early component's weaker influence compared to the more prominent Late component.

In summary, our findings reveal the critical role of both early sensory encoding and later decision-making stages in cross-modal associations. Cross-modal congruency primarily influenced the rate and timing of sensory evidence accumulation rather than altering the decision boundary, suggesting that congruency modulates how sensory information is collected rather than changing the threshold for decisions. Our results underscore the importance of modality in evidence accumulation and highlight the need for future studies to explore the sequential and interactional nature of EEG components across different sensory modalities. Understanding these mechanisms could inform more effective models of multisensory decision-making and expand our knowledge of how sensory congruency influences perceptual judgments.

## Data Availability

The raw data supporting the conclusions of this article will be made available by the authors, without undue reservation.
